# Lin28a uses distinct mechanisms of binding to RNA and affects miRNA levels positively and negatively

**DOI:** 10.1261/rna.059196.116

**Published:** 2017-03

**Authors:** Jakub Stanislaw Nowak, Fruzsina Hobor, Angela Downie Ruiz Velasco, Nila Roy Choudhury, Gregory Heikel, Alastair Kerr, Andres Ramos, Gracjan Michlewski

**Affiliations:** 1 Wellcome Trust Centre for Cell Biology, University of Edinburgh, Edinburgh, EH9 3BF, United Kingdom; 2 Institute of Structural and Molecular Biology, University College London, London, WC1E 6XA, United Kingdom

**Keywords:** miRNA-9, let-7, Lin28a, neuronal differentiation

## Abstract

Lin28a inhibits the biogenesis of let-7 miRNAs by triggering the polyuridylation and degradation of their precursors by terminal uridylyltransferases TUT4/7 and 3′-5′ exoribonuclease Dis3l2, respectively. Previously, we showed that Lin28a also controls the production of neuro-specific miRNA-9 via a polyuridylation-independent mechanism. Here we reveal that the sequences and structural characteristics of pre-let-7 and pre-miRNA-9 are eliciting two distinct modes of binding to Lin28a. We present evidence that Dis3l2 controls miRNA-9 production. Finally, we show that the constitutive expression of untagged Lin28a during neuronal differentiation in vitro positively and negatively affects numerous other miRNAs. Our findings shed light on the role of Lin28a in differentiating cells and on the ways in which one RNA-binding protein can perform multiple roles in the regulation of RNA processing.

## INTRODUCTION

Cell lineage abnormal 28a (Lin28a) is one of the best-studied proteins with respect to its role in the regulation of miRNA biogenesis. Lin28a is very well conserved across many species, and it was first described in *Caenorhabditis elegans*, in which mutations of the protein cause defects in developmental timing and accelerate the differentiation of several types of cells (Moss et al. 1997). It contains two highly conserved RNA-binding motifs forming cold-shock (CSD) and tandem zinc-finger (ZnF) domains, with 79%–90% homology at the amino acid level across vertebrates (Ouchi et al. 2014). These domains are present in a number of RNA-binding proteins (such as YBX2, FRGY2, or NCp7); however, Lin28 proteins are the only metazoan proteins to have both (Balzer and Moss 2007). Expression profiling in metazoans showed that Lin28a is abundantly expressed in the early stages of embryonic development, during which it inhibits the biogenesis of miRNAs from the let-7 family. Lin28a expression is gradually restricted with lineage progression, which allows de-repression of let-7 production in more developed and differentiated cells (Seggerson et al. 2002; Moss and Tang 2003; Darr and Benvenisty 2009; Van Wynsberghe et al. 2011). The main mechanism by which Lin28a inhibits let-7 biogenesis is based on its interaction with the conserved terminal loop (CTL) (Michlewski et al. 2008) of pre-let-7 (Wulczyn et al. 2007; Newman et al. 2008; Rybak et al. 2008). This event creates a platform for terminal uridylyltransferase 4 (TUT4) and other members from the TUT family, which catalyze the addition of a poly(U) tail to pre-let-7 (Hagan et al. 2009; Thornton et al. 2012). Polyuridylation ultimately results in pre-let-7 destabilization and a decrease of mature let-7 (Hagan et al. 2009; Heo et al. 2009). The degradation of poly(U) pre-let-7 is performed in the cytoplasm independently of the RNA exosome by 3′–5′ Dis3l2 exoribonuclease from the RNase II/RNB family (Chang et al. 2013; Ustianenko et al. 2013), which has a preference for unstructured and poly(U)-rich RNAs (Chang et al. 2013; Malecki et al. 2013; Munoz-Tello et al. 2015; Viegas et al. 2015). Moreover, polyuridylation of pre-let-7 precludes Dicer from generating mature let-7 (Heo et al. 2009). Other mechanisms for the control of let-7a that operate at the level of DGCR8/Drosha processing have also been suggested (Piskounova et al. 2008). Additionally, close homolog Lin28b also controls let-7 levels in vivo (Shyh-Chang and Daley 2013; Golden et al. 2015).

Recently, we have shown that during the early stages of neuronal differentiation, Lin28a controls the levels of neuro-specific miRNA-9 by destabilization of its precursor (Nowak et al. 2014). MiRNA-9 is an ancient miRNA whose origin extends back to the transition toward triploblasts (Wheeler et al. 2009). In higher vertebrates, miRNA-9 has been directly linked with neuronal development. A genome-wide profiling of miRNA classified miRNA-9 as a brain-enriched miRNA (Lagos-Quintana et al. 2002; Krichevsky et al. 2003; Landgraf et al. 2007). Furthermore, its expression profiling suggests that miRNA-9 is dynamically regulated throughout neuronal differentiation (Miska et al. 2004; Sempere et al. 2004). Expression of miRNA-9 is switched on during mid-embryogenesis after the development of the neuronal scaffold and is associated with active neurogenic areas (Darnell et al. 2006; Walker and Harland 2008; Coolen et al. 2012). MiRNA-9 is generally excluded from brain regions containing undifferentiated neuronal progenitors and from areas with late differentiation onset, such as the midbrain–hindbrain region and the retina (Leucht et al. 2008; La Torre et al. 2013). Moreover, REST and CREB regulate the transcription of miRNA-9 primary transcripts (Laneve et al. 2010). Previously we demonstrated that Lin28a binds to the CTL of pre-miRNA-9 and decreases the cellular levels of miRNA-9 during retinoic acid-mediated neuronal differentiation of mouse teratocarcinoma P19 cells. We also showed that the Lin28a-mediated destabilization of pre-miRNA-9 is poly(U)-independent. Furthermore, constitutive expression of GFP-tagged Lin28a reduced the levels of let-7a but not miRNA-9, whereas untagged Lin28a inhibited both miRNA-9 and let-7a, leading to impaired neuronal differentiation. These results suggested that there are at least two distinct mechanisms by which Lin28a triggers pre-miRNA degradation and that both depend on the RNA substrate. Finally, because miRNA-9 regulation takes place in the first days of neuronal differentiation, it is unknown if there are other Lin28a-regulated miRNAs with discrete spatio-temporal expression during cellular differentiation.

Here, we present molecular and biophysical evidence that Lin28a uses two distinct modes of binding to pre-let-7a and pre-miRNA-9, which could explain its alternative mechanisms of action. We reveal that 3′-5′ exoribonuclease Dis3l2 contributes to the regulation of miRNA-9 levels. Using small RNA-seq analysis of P19 cells with constitutive expression of Lin28a, we show that Lin28a controls production of many more miRNAs than previously recognized. We identified several miRNAs that are up-regulated by Lin28a overexpression. Importantly, our high-throughput results confirm the limited function of GFP-tagged Lin28a and show that untagged Lin28a inhibits the production of a number of brain-specific miRNAs, including miRNA-9. Our results provide evidence that Lin28a has both positive and negative roles in the regulation of miRNA production and uses distinct mechanisms of binding to RNA.

## RESULTS

### Lin28a uses different modes of binding to pre-miRNA-9 and pre-let-7

Undifferentiated cells do not produce let-7 or miRNA-9 due to post-transcriptional and transcriptional control, respectively (Nowak et al. 2014). Lin28a binds to pre-let-7 and triggers uridylation-dependent degradation (Heo et al. 2009; Chang et al. 2013; Ustianenko et al. 2013). In the course of neuronal differentiation, gradual reduction of Lin28a expression allows de-repression of let-7 biogenesis in more developed and differentiated cells (Seggerson et al. 2002; Moss and Tang 2003; Darr and Benvenisty 2009; Van Wynsberghe et al. 2011). Pri-miRNA-9 starts being produced at day 3 of differentiation but mature miRNA-9 only starts to accumulate from day 4. Previously we showed that Lin28a triggers uridylation-independent degradation of pre-miRNA-9 and regulates miRNA-9 levels in early stages of differentiation, or when misexpressed (Nowak et al. 2014). Furthermore, we have shown that prolonged expression of Lin28a results in defective retinoic acid-driven neuronal differentiation.

**FIGURE 1.**
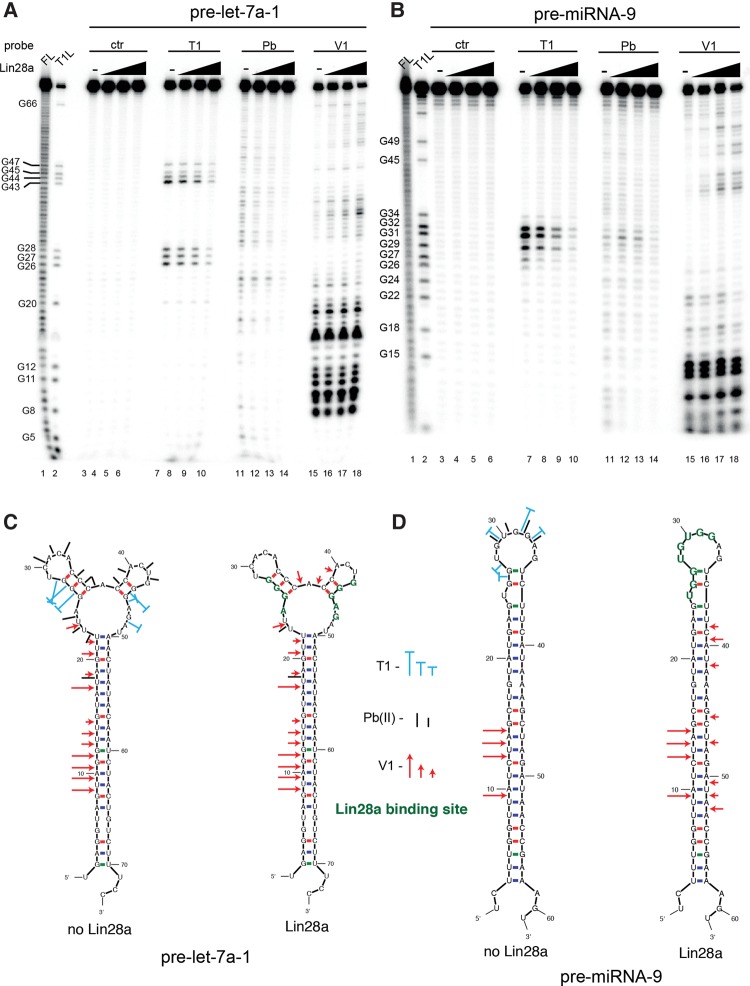
Footprinting analysis of the Lin28a interaction with pre-let-7a-1 and pre-miRNA-9. (*A*,*B*) Structure probing and footprint analysis of the pri-miRNA-9 and pre-let-7a-1 in complex with Lin28a. Cleavage patterns were obtained for 5′ ^32^P-labeled transcripts incubated in the absence (Lanes *3*–*6*) or in the presence of increasing amounts of recombinant Lin28a (Lanes *7*–*18*) (100 ng, 200 ng, 500 nM), treated with ribonuclease T1 (lanes *7*–*10*), Pb (II)-lead ions (lanes *11*–*14*), and ribonuclease V1 (lanes *15*–*18*). FL (lane *1*) and T1L (lane *2*) denote nucleotide residues subjected to partial digestion with formamide (every nucleotide) or ribonuclease T1 (G-specific cleavage). Electrophoresis was performed in a 12% polyacrylamide gel under denaturing conditions. The positions of the selected G residues are indicated. Nucleotides are numbered from the 5′ site of Drosha cleavage. (*C*,*D*) Proposed structures of free and Lin28a-bound pri-miRNAs. The sites and intensities of cleavage generated by structure probes are shown. The green nucleotides represent the nucleotides with the most significant Lin28a footprint.

To determine the differences between pre-miRNA-9/Lin28a and pre-let-7a/Lin28a complexes, we performed RNA structure probing with lead ions and T1 and V1 ribonucleases. With pre-let-7a-1, there was a significant Lin28a footprint around the well-known AGGG and GGAG Lin28a-binding motifs, which are located in the conserved terminal loop (Fig. 1A,C). These regions have been previously shown by structural studies to interact, respectively, with the cold-shock (CSD) and zinc-finger (ZnF) domains of Lin28a (Lightfoot et al. 2011; Nam et al. 2011; Desjardins et al. 2012; Loughlin et al. 2012). Binding of recombinant Lin28a resulted in increased cleavage by V1 ribonuclease with decreased activity of Pb (II) cleavage in the central region of the terminal loop, which suggests structural rearrangements of the pre-let-7a-1 structure (Fig. 1A,C). For pre-miRNA-9, the most significant Lin28a footprint was identified within the GU-rich region of its conserved terminal loop but not the GGAG motif (Fig. 1B,D). Similarly to pre-let-7a-1, association of Lin28a resulted in increased activity of V1 ribonuclease, which suggests higher-order structural rearrangements.

### Isolated CSD of Lin28a can bind to pre-miRNA-9 but not to pre-let-7

**FIGURE 2.**
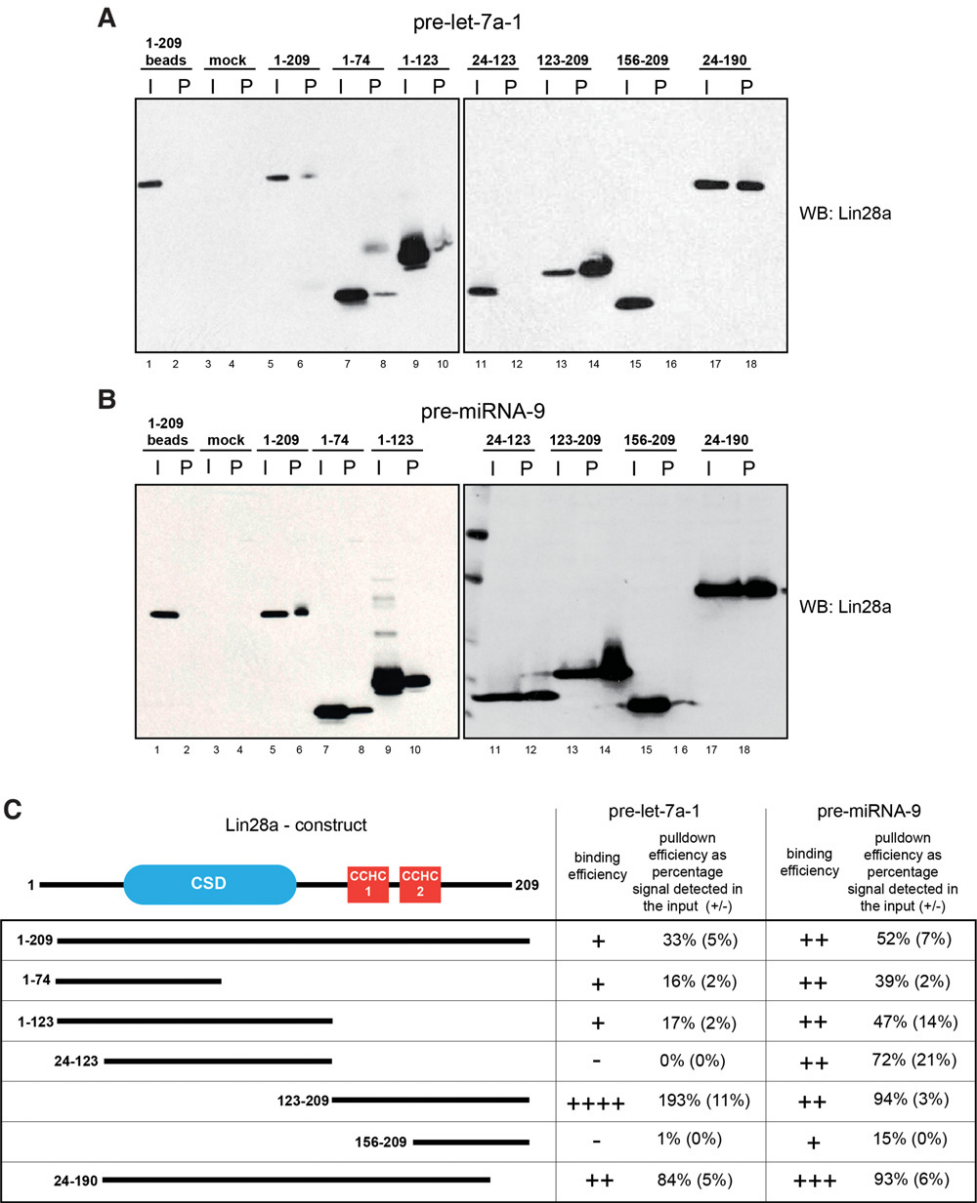
Lin28a binds to pre-miRNA-9 and pre-let-7a-1 using different domains. (*A*,*B*) Western blot analysis of pre-miRNA-9 and pre-let-7a-1 pull-downs with HeLa cell extracts overexpressing human Lin28a and its truncation mutants. Lanes with odd numbers represent 4% (100 µg) of the loading controls (I). Lanes with even numbers show the pull-down reactions (P). (*C*) Schematic representation of Lin28a truncations and relative binding efficiency, quantified as a percentage of the signal detected in the corresponding loading controls. The results are representative of at least three independent experiments (the value ranges from different experiments are shown in brackets).

To establish if pre-miRNA-9 interaction with Lin28a is indeed different compared to that observed in the case of pre-let-7a-1, we performed pull-down assays in HeLa cell extracts with various overexpressed, truncated forms of Lin28a (Fig. 2). Both pre-miRNA-9 and pre-let-7a-1 pulled down nearly full-length 1-209 and N and C termini truncated 24-190 Lin28a (Fig. 2A,B). Notably, pre-miRNA-9 pulled down CSD-containing peptide constructs 1-123 and 24-123 much more efficiently than pre-let-7a-1 (Fig. 2C). In particular, pre-let-7a-1 did not pull down the CSD-containing peptide 24-123 at all, whereas binding of this peptide to pre-miRNA-9 was detected at ∼72% of the signal from the loading control (Fig. 2C). This is in line with previous observations showing significantly weaker interaction of CDS with pre-let-7g when compared with the full-length Lin28a (Desjardins et al. 2012). At the same time, pre-let-7a-1 was able to pull down truncated Lin28a with ZnF domain (123–209) twice as efficiently as pre-miRNA-9 (Fig. 2C) and more efficiently than the full-length Lin28a. Surprisingly, Desjardins et al. (2012) showed a similar affinity of isolated ZnF domain and full-length Lin28a to pre-let-7g. This could be due to different accessibility of Lin28a-binding motifs in the terminal loops of pre-let-7a-1, pre-let-7g, and pre-miRNA-9. Two other constructs (1–74, 156–209) showed some degree of differential binding affinity between pre-miRNA-9 and pre-let-7a-1. These results strongly suggest that pre-miRNA-9 and pre-let-7a-1 bind Lin28a using different domains.

### GGAG motif is not responsible for Lin28a binding to pre-miRNA-9

**FIGURE 3.**
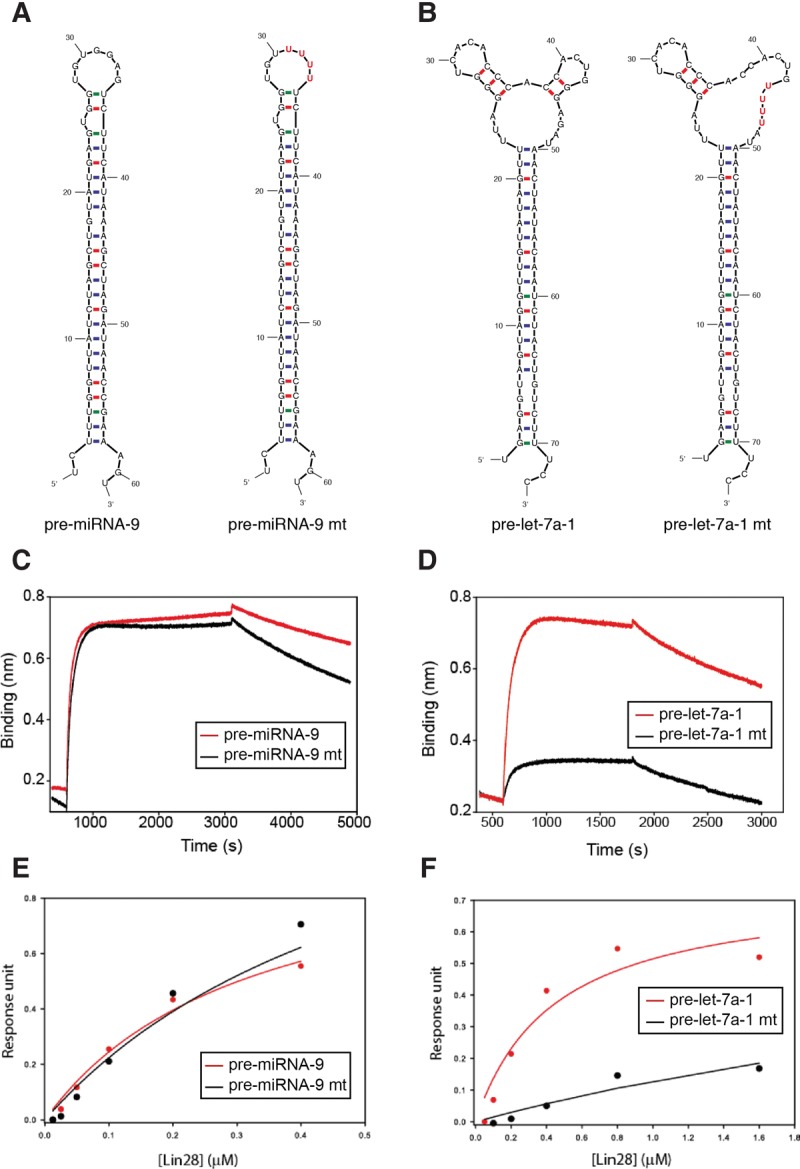
The two RNA binding domains of Lin28a play a different role in the recognition of pre-let-7a and pre-miRNA-9. (*A*,*B*) Secondary structures of wild-type and GGAG/UUUU pre-miRNA-9 and pre-let-7a-1 mutants (mt). Mutated residues are marked in red. (*C*,*D*) BLI data reporting on Lin28 binding to pre-miR-9 (wild-type and mutant) and pre-let-7-a (wild-type and mutant) at a protein concentration of 0.4 µM and 0.8 µM, respectively (*E*,*F*). The values of BLI signals at equilibrium upon exposing the immobilized RNAs to different concentrations of Lin28a are plotted against protein concentrations. The binding isotherms are also displayed. BLI data show that Lin28a binding to the pre-miRNA-9 RNA is only marginally affected by the mutation of the ZnF-specific sequence, whereas mutating the ZnF recognition sequence leads to a very significant drop in affinity for the pre-let-7a RNAs.

To compare the binding affinity of Lin28a for pre-miRNA-9 and pre-let-7a-1 and to investigate the role played by the ZnF and cold shock domains in RNA recognition in more detail, we designed an assay orthogonal to the pull downs described above. We disrupted the ZnF–RNA interactions by mutating the ZnF target RNA sequence (GGAG to UUUU) in the terminal loops of pre-miRNA-9 and pre-let-7a-1 (Fig. 3A,B) and used BioLayer Interferometry (BLI) to probe changes in RNA binding (Fig. 3C,D). We immobilized biotinylated pre-miRNA-9 and pre-let-7a-1 on streptavidin BLI sensors and assessed their interaction with recombinant Lin28a. Lin28a copurifies with nonspecific nucleic acid; therefore, our assays were effectively competition experiments rather than two-way component experiments. This showed that Lin28a binds both pre-miRNA-9 and pre-let-7a-1 with a *k*_d_ in the high nanomolar range (∼300 nM and ∼400 nM, respectively) (Fig. 3C,D). Previous reports of Lin28a dissociation constants with fragments from pre-let-7 substrates were in the range of 0.15 nM to 15 µM (Piskounova et al. 2008; Lightfoot et al. 2011; Nam et al. 2011; Desjardins et al. 2012). The differences most likely arise from presence or absence of RNA competitors in the binding buffers and different RNA substrates tested. Our experiments showed that mutation of the canonical ZnF binding site GGAG to UUUU led to a significant decrease in Lin28a binding to pre-let-7a-1 of more than 10-fold (mutant *k*_d_ is >>6 µM, Fig. 3D), but not in Lin28a binding to pre-miRNA-9 (*k*_d_ is still ∼300 nM, Fig. 3C). Importantly, GGAG/UUUU mutation did not abrogate inhibitory activity of Lin28a on pre-miRNA-9 in HeLa cells (Supplemental Fig. 1). Altogether, these results confirm that Lin28a recognizes the two RNAs in a different manner, with the CSD playing a more prominent role in the recognition of pre-miRNA-9 and the ZnF being essential in the recognition of pre-let-7a.

### EMSA with recombinant Lin28a validates BLI assays

**FIGURE 4.**
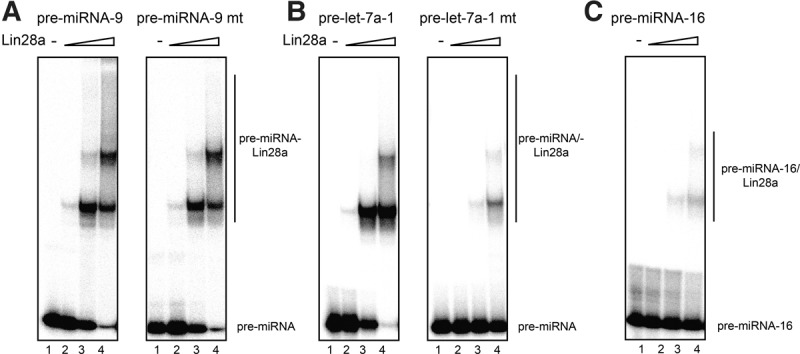
Lin28a EMSA with pre-let-7a and pre-miRNA-9 confirms BLI results. (*A*) EMSA analysis with pre-miRNA-9 and pre-miRNA-9 mt. Lane *1* represents the loading control. Lanes *2*–*4* show EMSA with increasing amount of Lin28a (50, 100, and 200 ng). (*B*) EMSA analysis with pre-let-7a-1 and pre-let-7a-1 mt. Lane *1* represents the loading control. Lanes *2–4* show EMSA with increasing amount of Lin28a (50, 100, and 200 ng). (*C*) EMSA analysis with pre-miRNA-16. Lane *1* represents the loading control. Lanes *2–4* show EMSA with increasing amount of Lin28a (50, 100, and 200 ng).

In order to validate BLI assays, we used EMSA with radiolabeled pre-miRNA probes and increasing amounts of recombinant Lin28a (Fig. 4). Both pre-miRNA-9 and pre-miRNA-9 mt were shifted by the Lin28a forming monomeric and multimeric complexes (Fig. 4A). In line with our BLI experiments, only wild-type pre-let-7a-1 but not pre-let-7a-1 mt was efficiently shifted by the Lin28a (Fig. 4B). The stepwise multimerization of Lin28a has been shown before and it is believed to be important for inhibition of Dicer cleavage (Desjardins et al. 2014). Importantly, pre-miRNA-16, which was shown by many groups not to bind Lin28a, does not shift Lin28a efficiently (Fig. 4C).

### Dis3l2 is involved in regulating levels of miRNA-9 during neuronal differentiation of P19 cells

**FIGURE 5.**
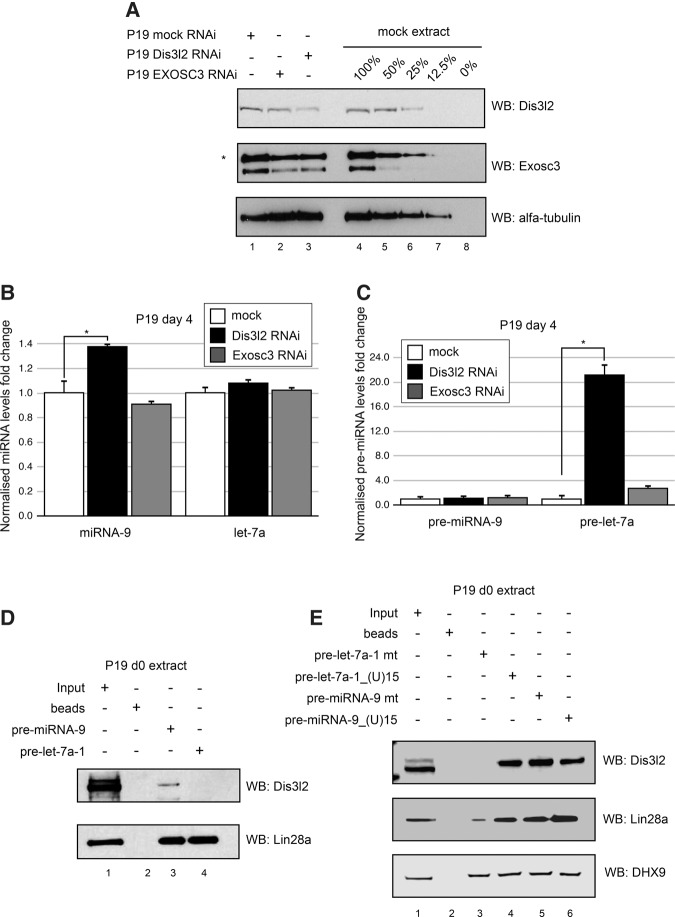
Dis3l2 affects miR-9 levels and binds pre-miR-9 in a poly(U)-independent manner. (*A*) Western blot analysis of protein extracts from mock-depleted P19 cells (Lane *1*), Dis3l2-depleted P19 cells (Lane *2*), and EXOSC3-depleted P19 cells (Lane *3*). Lanes *4*–*8* show serial dilutions of total protein extracts from mock-depleted P19 cells, providing an estimation of the linearity of the Western blot assay and the limit of detection. Real-time qRT-PCR analysis of the mature (*B*) and pre- (*C*) miRNA-9 and let-7a levels on day 4 of RA-induced neuronal differentiation. The results from the mock-depleted cells are shown as white bars; the results from Dis3l2-depleted cells are shown as black bars; the results from EXPSC3-depleted cells are shown as gray bars. The values were normalized to miR-16 levels. The fold change was plotted relative to values derived from mock-depleted cells, which were set to one. The mean and standard deviations (SD) of three independent biological replicates are shown. Statistical significance was calculated using a *t*-test; (*) *P* ≤ 0.05. (*D*) Western blot analysis of pre-miRNA pull-down with d0 P19 cell extracts for Dis3l2 and Lin28a. Lane *1* represents 4% (100 µg) of the loading control. Lane *2* shows the reaction with beads alone. Lanes *3* and *4* represent pre-miRNA-9 and pre-let-7a-1 pull-downs, respectively. The results are representative of at least three independent experiments. (*E*) Western blot analysis of pre-miRNA pull-down with d0 P19 cell extracts for Dis3l2, Lin28a, and DHX9. Lane *1* represents 4% (100 µg) of the loading control. Lane *2* shows the reaction with beads alone. Lanes *3*,*4*,*5*, and *6* represent pre-let-7a-1 mt, pre-let-7a-1_(U)15, pre-miRNA-9 mt, and pre-miRNA-9_(U)15 pull-downs, respectively. The results are representative of at least three independent experiments.

To determine whether RNA degradation enzymes can cooperate with Lin28a in the destabilization of pre-miR-9 during neuronal differentiation, we performed RNAi against Dis3l2 and Exosc3 (Fig. 5A). Exosc3 is an essential, noncatalytic component (Liu et al. 2006) of the RNA exosome (Mitchell et al. 1997), which plays a pivotal role in the binding and presentation of RNA for degradation. Dis3l2 and Exosc3 were depleted by ∼70% and 50%, respectively. Surprisingly, Dis3l2 knockdown also resulted in down-regulation of Exosc3. The miRNA levels were analyzed in P19 cells treated by siRNAs and subsequently differentiated until day 4 and were compared to reciprocal, mock-treated cells. At this stage, both pri-miRNA-9 and pri-let-7 are transcribed but Lin28a suppresses their processing. As previously reported, Dis3l2 knockdown had no significant effect on mature let-7 (Fig. 5B; Chang et al. 2013). This is mainly due to the pre-let-7a poly(U) tail, which inhibits Dicer processing (Heo et al. 2009). The same Dis3l2 knockdown resulted in a subtle but reproducible increase in miRNA-9 levels (Fig. 5B). It is important to note that expression of miRNA-9 only starts at day 3 of P19 cell neuronal differentiation (Nowak et al. 2014), hence the observed small changes in miRNA-9 expression. Additionally, due to Dicer inability to process uridylated pre-let-7a it was stabilized 20-fold in Dis3l2 knockdown (Fig. 5C). Conversely, levels of pre-miRNA-9 were unaffected by Dis3l2 depletion. Unconstrained Dicer processing of the stabilized pre-miRNA-9 could explain the lack of stabilization of pre-miR-9 upon Dis3l2 knockdown*.* The Exosc3 knockdown had no effect on the levels of either mature miRNA-9 or let-7 (Fig. 5B) but resulted in threefold up-regulation of pre-let-7 (Fig. 5C). These results suggest that Dis3l2, but not the RNA exosome, plays a role in the down-regulation of miRNA-9 levels.

To determine whether Dis3l2 interacts with pre-miRNA-9 in a poly(U)-independent manner, we performed RNA pull-down assays in extracts derived from undifferentiated P19 cells (Fig. 5D). To see if the interaction was specific, we used pre-let-7a-1 as a control because it was previously reported to require a poly(U) tail for efficient Dis3l2 binding (Chang et al. 2013). Our chemical coupling method of RNA to agarose beads via 3′ ribose protects the RNA from 3′ uridylation. As described before (Nowak et al. 2014), both pre-miRNA-9 and pre-let-7a-1 interacted with Lin28a (Fig. 5D). However, only pre-miRNA-9 pulled down Dis3l2 in a uridylation-independent manner. This is surprising as Dis3l2 was shown to bind 3′ ends of RNAs with preference toward multiple U residues (Faehnle et al. 2014). Furthermore, pre-let-7a_(U)15, pre-miRNA-9_(U)15, and pre-miRNA-9 mt, but not pre-let-7a-1 mt, pulled down Dis3l2 with similar efficiency (Fig. 5E). We also noted that pre-miRNA-9 mt shows increased binding to Dis3l2 (Fig. 5E) compared to pre-miRNA-9 (Fig. 5D). This could be due to the fact that the pre-miRNA-9 mt has additional U residues in the terminal loop, which might have created a Dis3l2 binding site. Altogether, these results indicate that complexes between Lin28a, Dis3l2, and pre-miRNAs can be formed even in the absence of a poly(U) tail and that the complexes formed by pre-miRNA-9 and pre-let-7a are different.

### Dis3l2 destabilizes pre-miRNA-9 in vitro

**FIGURE 6.**
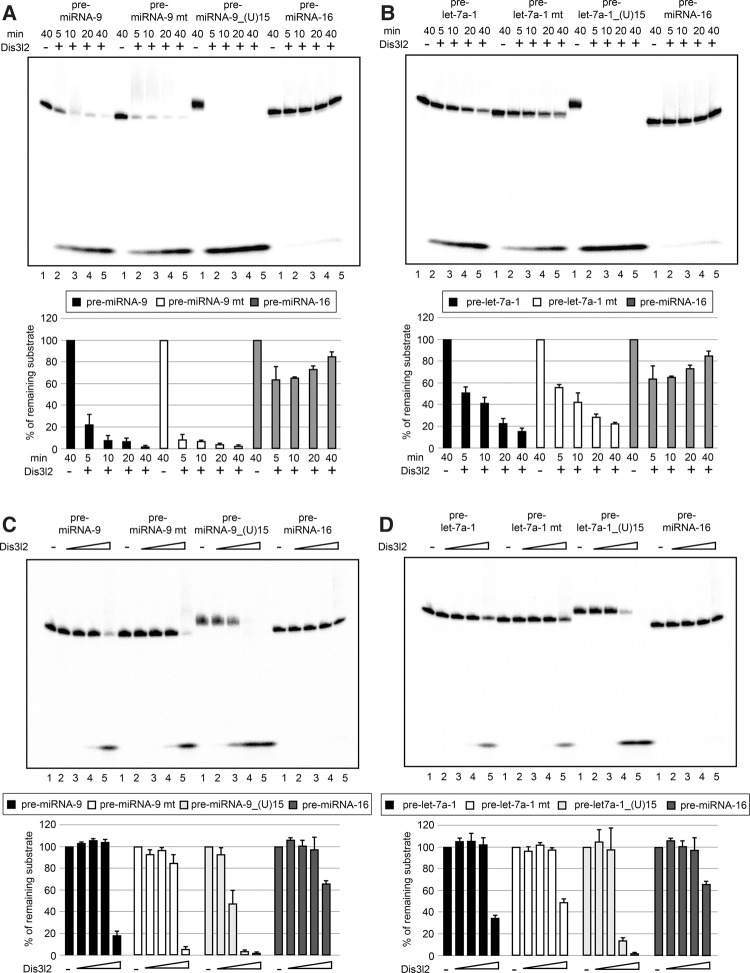
Dis3l2 destabilizes pre-miRNA-9 in vitro. Internally radiolabeled pre-miRNA-9, pre-miRNA-9 mt, pre-miRNA-9_(U)15, and pre-miRNA-16 (*A*), pre-let-7a-1, pre-let-7a-1 mt, pre-let-7a-1_(U)15, and pre-miRNA-16 (*B*) (3 × 10^3^ c.p.m. [counts per minute], ∼6 pmol) were incubated in the buffer only for 40 min (Lane *1*). Where indicated, 200 ng of recombinant Dis3l2 proteins were added to the reaction, which were run for 5, 10, 20, and 40 min. The products were analyzed on an 8% denaturing polyacrylamide gel. The results are representative of at least three independent experiments. The graphs represent quantification of the substrate's intensities. The values were plotted relative to the control reactions set to 100. The mean and standard deviations (SD) of three independent experiments are shown. Internally radiolabeled pre-miRNA-9, pre-miRNA-9 mt, pre-miRNA-9_(U)15, and pre-miRNA-16 (*C*), pre-let-7a-1, pre-let-7a-1 mt, pre-let-7a-1_(U)15, and pre-miRNA-16 (*D*) (3 × 10^3^ c.p.m. [counts per minute], ∼6 pmol) were incubated for 10 min with an increasing amount of Dis3l2 (0.2, 2, 20, 200 ng). The products were analyzed the same as described above.

Previously, we showed that pre-miRNA-9 is destabilized at the early stages of P19 cell neuronal differentiation (Nowak et al. 2014). To determine whether Dis3l2 is directly responsible for the role in pre-miRNA-9 degradation, we performed in vitro cleavage assays using recombinant Dis3l2 (Lubas et al. 2013). For pre-miRNA-9 and pre-miRNA-9 mt, addition of recombinant Dis3l2 resulted in robust time and concentration-dependent RNA degradation (Fig. 6A,C). Dis3l2 did not affect the control pre-miRNA-16 in similar conditions (Fig. 6A–D). At the same time, the artificial pre-miRNA-9_(U)15 and pre-let-7a-1_(U)15 were fully degraded after 5 min of incubation, confirming that Dis3l2 prefers U-tailed substrates (Fig. 6A,B). Importantly, Dis3l2 activity on the pre-let-7a-1 and pre-let-7a-1 mt substrates was markedly lower when compared to pre-miRNA-9 (Fig. 6B). For example, after 10 min of incubation we recovered <10% of pre-miRNA-9 substrate and >40% of pre-let-7a-1 substrate (Fig. 6A,B). Furthermore, Dis3l2 titration revealed that pre-miRNA-9_(U)15 is degraded more efficiently than pre-let-7a-1_(U)15 (Fig. 6C,D). Altogether, these results demonstrate that pre-miRNA-9 RNA is a good substrate for Dis3l2. Surprisingly, addition of recombinant Lin28a slowed down Dis3l2-mediated degradation of pre-miRNA-9 and pre-let-7a-1 (Supplemental Fig. 2). This might be a consequence of a lack of eukaryotic-specific protein modifications or absence of additional, yet uncharacterized cofactors. Thus, it remains to be established how Dis3l2 cooperates with cofactors, such as Lin28a, whose depletion leads to pre-miRNA-9 stabilization (Nowak et al. 2014). In summary, these results show that Dis3l2 could be directly involved in the degradation of pre-miRNA-9.

### Constitutive expression of untagged Lin28a deregulates the levels of many miRNAs during neuronal differentiation of P19 cells

**FIGURE 7.**
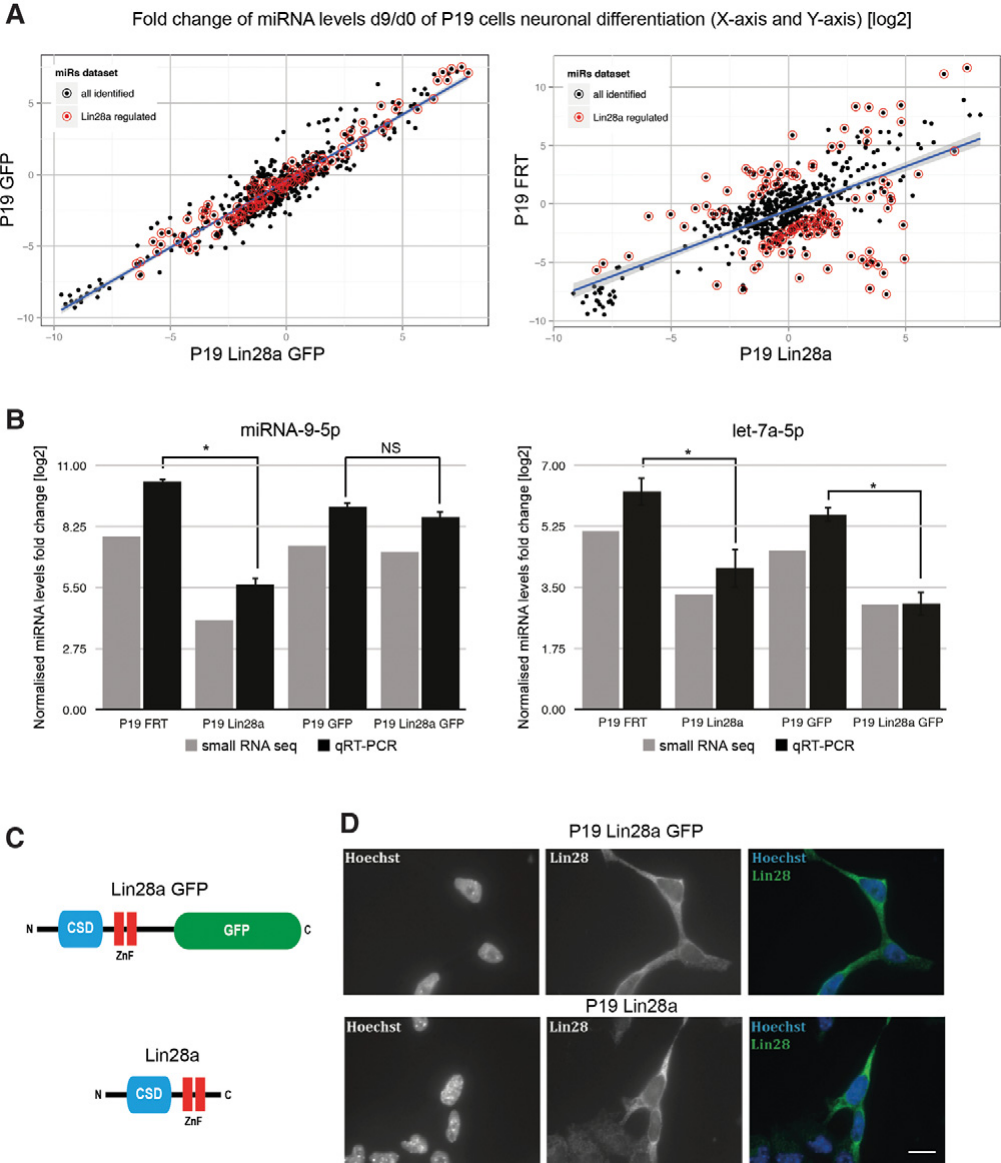
Constitutive expression of untagged Lin28a affects the levels of numerous miRNAs during RA-induced neuronal differentiation of P19 cells. (*A*) Scatterplots of the fold change between day 0 and day 9 of the neuronal differentiation of P19 cells with GFP-tagged Lin28a and GFP only (*left* graph) and of P19 cells with untagged Lin28a and the control cell line (*right* graph). MiRNAs regulated more than twofold up or down by untagged Lin28a but not regulated by GFP-tagged Lin28a are highlighted with red circles. (*B*) Real-time qRT-PCR analysis of mature miRNA-9 and let-7a represented by fold change between d0 and d9. The values were normalized to miRNA-16 levels. The fold change was plotted relative to values derived from undifferentiated cells (d0), which were set to one. The mean and standard deviations (SD) of three independent biological replicates are shown. Statistical significance was calculated using a *t*-test; (*) *P* ≤ 0.05. (NS) Statistically nonsignificant. (*C*) Schematic representation of Lin28a tagged with GFP and wild-type Lin28a. (*D*) Immunofluorescence staining of Hoechst (blue), Lin28a (green) in P19 cells showing localization of both untagged and GFP-tagged Lin28a predominantly in the cytoplasm. Scale bar, 10 µm.

Our previous findings demonstrated that prolonged expression of Lin28a impairs neuronal differentiation and miRNA-9 biogenesis (Nowak et al. 2014). Here, to determine which additional miRNAs are misexpressed upon constitutive Lin28a expression, we performed small RNA sequencing in samples derived from undifferentiated (day 0, d0) and differentiated (day 9, d9) control P19 cells and cells that constitutively express GFP-tagged (at the C terminus) or untagged Lin28a (Fig. 7), as previously described (Nowak et al. 2014). We compared the expression level changes of mature miRNAs, represented by the d9/d0 ratio, in P19 Lin28a and P19 Lin28a GFP cells to the changes in the reciprocal untargeted P19 FRT and P19 GFP control cell lines. We observed that the constitutive expression of untagged Lin28a but not of GFP-tagged Lin28a had a profound impact on the levels of many mature miRNAs during P19 cell neuronal differentiation, including miRNA-9 (Fig. 7A,B). Interestingly, other brain-enriched miRNAs, such as miRNA-124 and miRNA-138, were also negatively affected by Lin28a expression (Supplemental Fig. 3). Due to its impaired function, the effects of the constitutive expression of GFP-tagged Lin28a were much more modest and extended to fewer miRNAs (Fig. 7A), including let-7a (Fig. 7B).

To further analyze our data, we selected miRNAs that were regulated more than twofold by the constitutive expression of untagged Lin28a but were not affected (less than a twofold change) by GFP-tagged Lin28a (Fig. 7A; Supplemental Fig. 3). Apart from the 54 miRNAs that were down-regulated by two- to sixfold, we observed 110 miRNAs that were up-regulated from two- to 95-fold by the constitutive expression of Lin28a but not GFP-Lin28a (Supplemental Fig. 3). This implies that Lin28a can negatively and positively impact the production of many miRNAs. Importantly, miRNA-9 was one of the most down-regulated miRNAs by untagged Lin28a, which corroborates our previous findings (Nowak et al. 2014). To validate the small RNA-seq results, we measured the levels of selected miRNAs by qRT-PCR. The levels of miRNA-9 were significantly suppressed by constitutive Lin28a expression only (Fig. 7B), whereas let-7a expression was suppressed by both untagged and GFP-tagged Lin28a (Fig. 7B). Our previous data showed that the differences between untagged and GFP-tagged Lin28a do not arise from different protein levels (Nowak et al. 2014). Now we extend this to show that both untagged and GFP-tagged Lin28a (Fig. 7C) have identical cytoplasmic localization (Fig. 7D). This is in line with previous studies, which showed predominantly cytoplasmic localization of Lin28a (Moss and Tang 2003; Balzer and Moss 2007; Balzer et al. 2010). This reinforces the notion that the presence of the GFP tag interferes with Lin28a function on some miRNA precursors, such as miRNA-9, but not on others, such as let-7a. Lin28a crystal structure shows the C terminus extending toward CSD (Nam et al. 2011). Thus, we speculate that the GFP tag, which is placed at the C terminus of Lin28a, can interfere with CSD binding. This also agrees with our biochemical observations about different structural arrangements of pre-miRNA-9/Lin28a and pre-let-7a/Lin28a complexes.

### Forced expression of Lin28a results in up-regulation of many miRNAs

**FIGURE 8.**
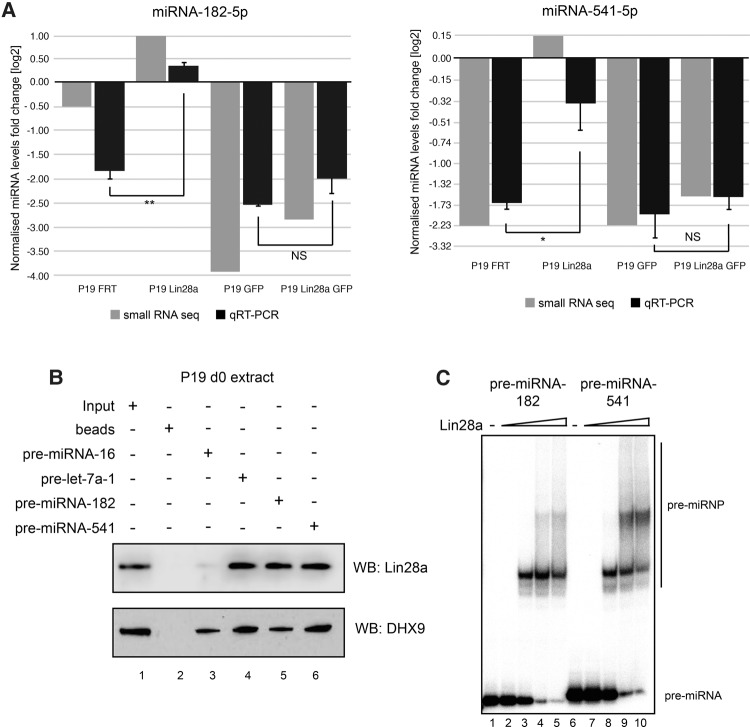
Constitutive expression of untagged Lin28a up-regulates miRNA-541 and miRNA-182 during RA-induced neuronal differentiation of P19 cells. (*A*) Real-time qRT-PCR analysis of mature miRNA-541-5p and miRNA-182-5p represented by fold change between d0 and d9. The values were normalized to miRNA-16 levels. The fold change was plotted relative to values derived from undifferentiated cells (d0), which were set to one. The mean and standard deviations (SD) of three independent biological replicates are shown. Statistical significance was calculated using a *t*-test; (*) *P* ≤ 0.05, (**) *P* ≤ 0.005. (*B*) Western blot analysis of pre-miRNA pull-down with d0 P19 cell extracts for Lin28a and DHX9. Lane *1* represents 4% (100 µg) of the loading control. Lane *2* shows the reaction with beads alone. Lanes *3*,*4*,*5*, and *6* represent pre-miRNA-16, pre-let-7a-1, pre-miRNA-182, and pre-miRNA-541 pull-downs, respectively. (*C*) EMSA analysis with pre-miRNA-182 and pre-miRNA-541. Lane *1* represents the loading control. Lanes *2*–*5* and *7*–*10* show EMSA with recombinant Lin28a (0.5, 5, 50, and 100 ng).

Surprisingly, levels of some miRNAs were elevated by constitutive expression of untagged Lin28a and remained relatively unchanged in the GFP-tagged Lin28a P19 cell line (Fig. 7; Supplemental Fig. 3). For validation we chose miRNA-182 and miRNA-541 as they represented miRNAs up-regulated by untagged Lin28a but not by GFP-tagged Lin28a. We validated the expression of miRNA-182 and miRNA-541 by qRT-PCR and observed that their levels were indeed higher in the presence of constitutively expressed Lin28a (Fig. 8A). Importantly, both pre-miRNA-182 and pre-miRNA-541 were able to pull down Lin28a from day 0 P19 cell extracts with similar efficiency to pre-let-7a-1 (Fig. 8B), whereas pre-miRNA-16 did not pull down Lin28a. Furthermore, Lin28a efficiently shifted both pre-miRNA-182 and pre-miRNA-541 in EMSA (Fig. 8C). However, transient Lin28a depletion in undifferentiated P19 cells did not result in a significant change in the levels of mature miRNA-182 and miRNA-541 (Supplemental Fig. 4). This suggests the existence of additional mechanism(s) safeguarding their biogenesis in undifferentiated cells. Alternatively, the positive effects on miRNA levels could be indirect. The exact mechanism(s) underlying the Lin28a-mediated up-regulation of miRNAs have yet to be determined.

### Lin28a binding sites are enriched in primary transcripts of Lin28a-affected miRNAs

**FIGURE 9.**
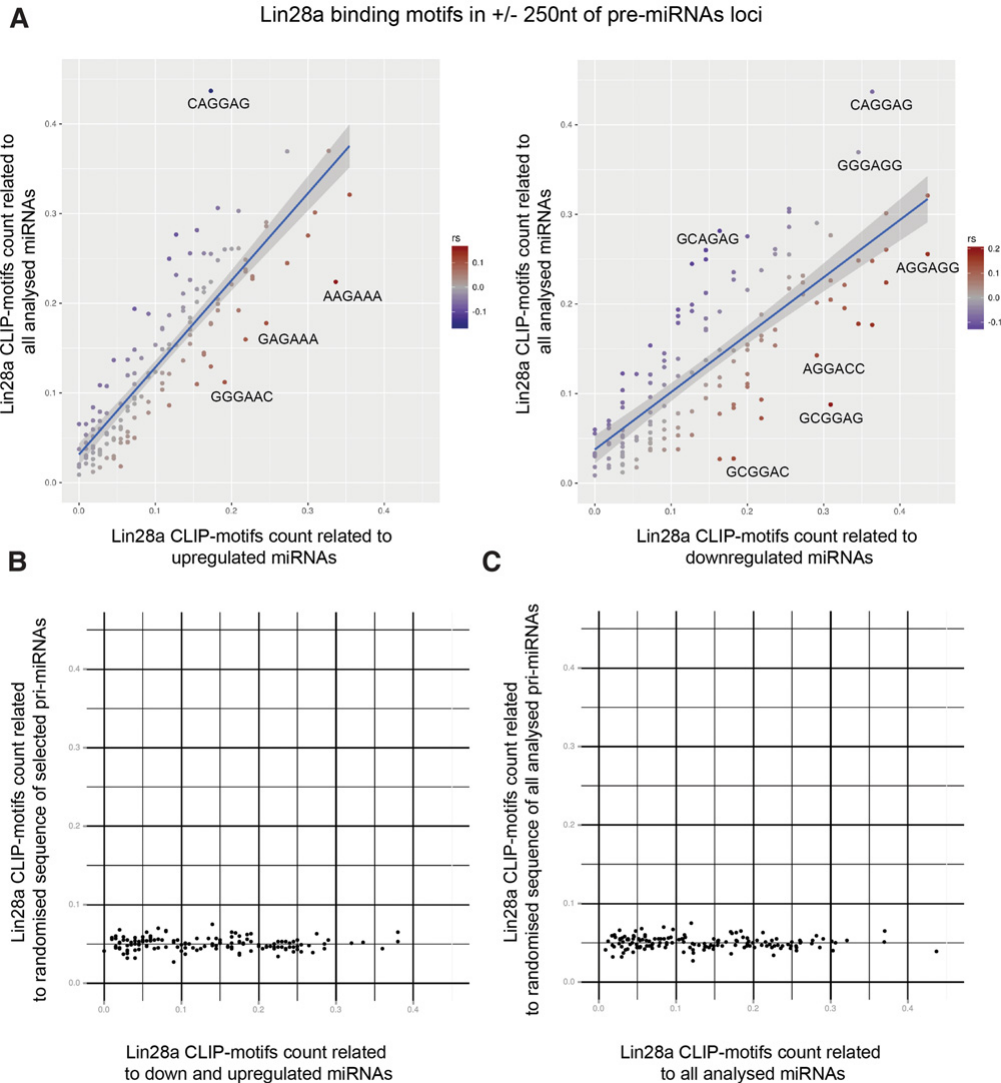
(*A*) Distribution of Lin28a CLIP motifs in loci of up-regulated (*left* panel) and down-regulated (*right* panel) miRNAs (miRNAs regulated more than twofold up or down by untagged Lin28a but not regulated by GFP-tagged Lin28a) versus all pri-miRNAs. (rs) Spearman's rank-order coefficient. (*B*,*C*) Correlation of Lin28a CLIP motifs count between (*B*) randomized sequence and selected (up- and down-regulated) pri-miRNAs; (*C*) randomized RNA sequence and all pri-miRNAs.

Finally, to determine if Lin28a binding motifs, previously revealed by CLIP analysis (Wilbert et al. 2012), are present in the primary transcripts of Lin28a-affected miRNAs (miRNAs regulated more than twofold up or down by untagged Lin28a but not regulated by GFP-tagged Lin28a), we performed bioinformatics analysis on a 500-nt sequence window surrounding the analyzed pre-miRNAs. We found that several Lin28a CLIP-motifs, including AAGAAA, GAGAAA, and GGGAAC, were enriched in proximity to the miRNAs up-regulated by Lin28a (Fig. 9A); whereas other motifs, including AGGAGG, GCGGAG, and GCGGAC, were enriched in proximity to the miRNAs down-regulated by Lin28a (Fig. 9A). Intriguingly, precursors of both miRNA-182 and miRNA-541, which were up-regulated by Lin28a, have AGAA motifs within their stems (Supplemental Fig. 4). Notably, different CLIP-motifs, including CAGGAG, were depleted from both up- and down-regulated miRNAs (Fig. 9A). These findings indicate that Lin28a might exert different mechanisms depending on the sequences to which it binds (Nowak et al. 2014). To determine whether the distribution of the Lin28a-CLIP motifs is significant, we randomized the 500-nt sequence windows surrounding the Lin28a up- and down-regulated and all analyzed miRNAs (Fig. 9A,B). Both sets of randomized pri-miRNA sequences showed no enrichment of Lin28a-CLIP motifs, which suggests that there is a selective pressure to keep Lin28a binding motifs in proximity to the miRNA loci and that the role of Lin28a or other protein(s) that use similar binding motifs in miRNA biogenesis could be more systemic and widespread. In summary, these results suggest that functional differences in Lin28a's mode of action may depend on the nature of its molecular interactions with the miRNA progenitor transcripts.

## DISCUSSION

At least three independent genome-wide studies have shown a broad range of Lin28a targets (Cho et al. 2012; Wilbert et al. 2012; Hafner et al. 2013). In the majority of cases, Lin28a was shown to interact with mRNA, whereas snoRNA and miRNA were minor targets (Hafner et al. 2013). However, these studies concentrated on RNA–protein interactions either in undifferentiated embryonic stem cells or differentiated, well-established cell models, such as HEK293 (Cho et al. 2012; Wilbert et al. 2012). In our study, we showed that constitutively expressed Lin28a regulates production of many other miRNAs during the retinoic acid-driven neuronal differentiation of mouse P19 cells. More miRNAs were up-regulated than down-regulated, suggesting that Lin28a exerts a positive role in miRNA production. Importantly, it remains to be elucidated whether these results were achieved by direct or indirect mechanisms. This could be done for example with newly identified small molecule inhibitors of Lin28a (Lim et al. 2016; Roos et al. 2016) in cellular systems as well as using in vitro binding assays. That said, many pre-miRNAs affected by Lin28a, such as pre-miRNA-9, -34c, or -181a, have already been shown to be good substrates for Lin28a binding (Towbin et al. 2013). Under physiological conditions, Lin28a is predominantly expressed at the early stages of cellular differentiation; therefore, for miRNAs to be affected by Lin28a they must be coexpressed at this stage. Both Lin28a and Lin28b are misexpressed in a number of tumor and cancer cells (Thornton and Gregory 2012; Zhou et al. 2013). It is now evident that Lin28a is an important oncogene in tumorigenesis (Tu et al. 2015) and an emerging maker of cancer stem cells (Ma et al. 2014). For example, prolonged expression of Lin28a in primitive mesenchymal kidney cells resulted in increased cell proliferation and Wilms’ tumor formation (Feng et al. 2012), which strongly suggests that Lin28a-mediated regulation of miRNA production can transcend the niche of undifferentiated cells and affect other miRNAs that are important for proper developmental timing. Thus, studying the systems where Lin28a is overexpressed is of utmost importance to understand its various roles in cancer biology. Interestingly, Dis3l2, which plays an important role in the Lin28a/let-7a pathway, is frequently mutated in Wilms’ tumor and causes the Perlman syndrome of overgrowth (Astuti et al. 2012; Reis et al. 2013).

In our previous work, we observed a substantial delay between the pri-miRNA-9 expression and the production of mature miRNA-9 during the neuronal differentiation of P19 cells. We also showed that Lin28a plays a role in restricting miRNA-9 production to later stages of neuronal differentiation (Nowak et al. 2014). The mechanism that triggers pre-miRNA-9 degradation, which is similar to pre-let-7a-1, is dependent on the conserved terminal loop but, unlike pre-let-7, is poly(U)-independent. In the case of pre-let-7, Lin28a binding attracts TUT4 and TUT7, which catalyze the addition of a poly(U) tail to its 3′ end (Hagan et al. 2009; Thornton et al. 2012) and subsequent degradation by Dis3l2 (Chang et al. 2013; Ustianenko et al. 2013), whereas binding of Lin28a to pre-miRNA-9 results in poly(U)-independent degradation (Nowak et al. 2014). Interestingly, both pre-miRNA-9 and pre-let-7a have the canonical Lin28a binding GGAG motif in their terminal loops. Our previous results showed that when the GGAG motif is present in a very small synthetic terminal loop it does not bind Lin28a (Choudhury et al. 2014). Hence, we propose that due to its structural architecture, pre-miRNA-9 predominantly interacts with Lin28a through its CSD domain, unlike in the case of pre-let-7, where both ZnF and CSD are involved in binding to the large terminal loop (Nam et al. 2011; Loughlin et al. 2012). Lin28 CSD binds with high affinity to single-stranded nucleic acids but with limited sequence specificity (Mayr et al. 2012). In the terminal loops of pre-let-7, it binds to GNGAY consensus sequence (Y, pyrimidine; N, any base) (Nam et al. 2011). Surprisingly, the CSD of YBX2 protein binds the well-defined AACA(A/U)C motif (Ray et al. 2013). Previously, it was shown that discrete structural and sequence differences in human pre-let-7a-3 (and its murine ortholog pre-let-7c-2) prevent Lin28a binding and bypass Lin28a-mediated inhibition (Triboulet et al. 2015). Furthermore, yeast three-hybrid analysis revealed that pre-let-7 transcripts bind using both CSD and ZnF, but other pre-miRNAs, such as pre-miRNA-152 or pre-miRNA-302d, bind Lin28a using CSD predominantly (Balzer et al. 2010). Altogether, we speculate that differential binding of Lin28a could lead to distinct pre-miRNP complex formation.

Pre-miRNA-9 characteristics allow efficient binding of both Lin28a and Dis3l2 in a poly(U)-independent manner (Fig. 5D). Moreover, recombinant Dis2l3 was able to efficiently cleave pre-miRNA-9 in vitro (Fig. 6A). However, synthetically polyuridylated pre-miRNA-9 is a better substrate for Dis3l2 (Fig. 6A). This agrees with previous results that Dis3l2 prefers uridylated substrates but can degrade many other transcripts (Lubas et al. 2013; Malecki et al. 2013). Dis3l2 RNAi resulted in moderate but highly reproducible up-regulation of miRNA-9 during early differentiation of P19 cells (Fig. 5B) but did not influence steady-state pre-miRNA-9 levels (Fig. 5C). We speculate that upon Dis3l2 knockdown, pre-miRNA-9 could be stabilized and thus provide more substrate for Dicer cleavage, which would generate more mature miRNA-9. So far there are no reports of direct interaction between Lin28a and Dis3l2. Notably, Lin28a is well known to recruit TUT4 to pre-let-7; however, there is no evidence of physical interactions between Lin28a and TUT4. Instead, it is suggested that Lin28a functions as a TUT4 processivity factor (Yeom et al. 2011). In the future it will be important to test if the same could be true for Dis3l2. A recent report has shown that Dis3l2 is involved in degradation of miRNAs, which are bound by highly complementary target RNAs (Haas et al. 2016). Further in-depth characterization of the pre-miRNA-9/protein complex is required to reveal the fine details of this interaction.

Lin28a is important for neuronal differentiation (Rybak et al. 2008; Balzer et al. 2010). Here, we show that its prolonged expression in differentiating cells positively and negatively affects numerous miRNAs. Furthermore, we present evidence that small differences in RNA secondary structures, such as those seen between the stem–loops of pre-let-7a and pre-miRNA-9, could determine the mode of RNA-binding protein interaction and RNP function. In summary, our results increase understanding regarding the ways in which RNA–protein interactions control RNA metabolism in cells and provide a framework for future analysis of physiologically important RNP complexes.

## MATERIALS AND METHODS

### Stable cell line generation

P19 cell lines with stable Lin28a-GFP or GFP-only expression were gifts from Dr. Eric Moss (Rutgers School of Biomedical and Health Sciences, formerly The University of Medicine and Dentistry, New Jersey, USA) (Balzer et al. 2010). Both lines were maintained under standard culture conditions. A P19 cell line expressing untagged Lin28a was developed using the Flp-in system (Life Technologies), according to the manufacturer's instructions and as previously described (Nowak et al. 2014).

### Cell culture and neuronal differentiation conditions

Mouse teratocarcinoma P19 cells and HeLa cells were grown in standard DMEM medium (Life Technologies) supplemented with 10% FBS (Life Technologies). All-trans retinoic acid (RA) (Sigma-Aldrich) was used to induce neuronal differentiation. In short, ∼12 × 10^6^ cells were plated on a nonadhesive dish in DMEM supplemented with 5% serum and with 1 µM RA. This induced the formation of embryonic bodies. After 4 d, the embryonic bodies were seeded in 10% FBS DMEM on an adhesive dish. Differentiation was followed up to 9 d post-induction. Plasmids encoding truncated Lin28a constructs were based on the pCG-T7-Lin28a construct as previously described (Michlewski and Caceres 2010) and were prepared using inverted PCR. Plasmids were transfected into HeLa cells using Lipofectamine 2000 reagent, as previously described (Choudhury et al. 2014).

### Immunofluorescence

Lin28a was visualized in P19 cells using primary monoclonal rabbit polyclonal anti-Lin28a (A177) (Cell Signaling Technology). Prior to microscopy, cells were plated on cover slips coated with 2 mL of 10 µg/mL PDL (Sigma-Aldrich P4707). At 24 h after plating, the cells were washed with PBS and fixed with 4% formaldehyde (Sigma-Aldrich 37% w/v in H2O 252549-500) for 10 min at RT. Next, the cells were permeabilized for 10 min at RT by adding 0.2% Triton-X (Sigma-Aldrich T9284-100). Subsequently, the cells were blocked for 15 min at RT with goat serum and incubated for 1 h at RT with primary antibody at a 1/1000 dilution in goat serum and for 1 h at RT with Alexa Fluor goat anti-rabbit 568 secondary antibody (Molecular Probes A-11036) at a dilution of 1/1000 in goat serum. In the last step, cells were counterstained with Hoechst dye (1/20,000) for 15 min at RT and mounted on slides using 15 µL of mounting medium (Molecular Probes Prolong Gold AntiFade P36930). Each of the above steps was separated by three washes with PBS for 5 min at RT. Mounted cells were visualized using a Zeiss Axio Imager Z1 fluorescent microscope.

### MiRNA qRT-PCR analysis

MiRNA qRT-PCR analysis was performed using the miScript qRT-PCR kit (Qiagen) on total RNA isolated with TRIzol reagent (Life Technologies), and each sample was run in duplicate. To assess the levels of the corresponding microRNAs, values were normalized to miRNA-16. For each measurement, three independent experiments were performed.

### Small RNA sequencing

Total cell RNA was extracted with TRIzol reagent and was subjected to quality control (QC) for SOLEXA sequencing (BGI Genomics). After a positive QC result, RNA was run on a PAGE gel, and species below 30 nt were extracted and ligated to SOLEXA adaptors at the 5′ and 3′ ends. Small RNA molecules were amplified for 17 cycles using PCR primers against SOLEXA adaptors, and fragments of ∼90 bp (small RNA + adaptor) were gel-purified and used directly for cluster generation and sequencing analysis using an Illumina Genome Analyzer. The image files generated by the sequencer were then processed to produce digital-quality data. The raw data were processed to generate clean reads by masking the adaptor sequences and removing contaminated reads (rRNA, tRNA, mRNA). Clean reads were mapped with zero-matches allowance onto a reference mouse genome using BGI-designed SOAPaligner software to locate each read on the genome sequence (Li et al. 2008). Subsequent annotation was performed using information in miRBase.

### Western blot analysis

Total protein samples (100 µg per lane) were run on 4%–12% NuPAGE SDS-PAGE electrophoresis with MOPS running buffer (Life Technologies) and were transferred onto a nitrocellulose membrane. The membrane was blocked overnight at 4°C with 1:10 Western Blocking Reagent (Roche) in TBS buffer with 0.1% Tween-20–TBST. The next day, the membrane was incubated for 1 h at RT with primary antibody solution in 1:20 Western Blocking Reagent diluted in TBST: rabbit polyclonal anti-Lin28a (A177) (1:1000, Cell Signaling Technology), rabbit polyclonal anti-Dis3l2 (1:1000, a kind gift from Andrzej Dziembowski), rabbit polyclonal anti-Exosc3 (1:2000, Abcam), and mouse-monoclonal anti-β-tubulin (1:10,000, Sigma-Aldrich), and rabbit polyclonal anti-DHX9 (1:1000, Abcam). After washing in TBST, the blots were incubated with the appropriate secondary antibodies conjugated to horseradish peroxidase and were detected with SuperSignal West Pico detection reagent (Thermo Scientific). The membranes were stripped using ReBlot Plus Strong Antibody Stripping Solution (Chemicon) equilibrated in water, blocked in 1:10 western blocking solution in TBST and reprobed, as described above.

### Electrophoretic mobility shift assay

Electrophoretic mobility shift assays (EMSA) were performed with internally labeled pre-miRNA transcript and proteins produced in *Escherichia coli*. Gel-purified probes (50 × 10^3^ c.p.m. [counts per minute], ∼20 pmol) were incubated in 15-µL reaction mixtures containing the indicated amounts of proteins in Roeder D buffer (100 mM KCl, 20% [v/v] glycerol, 0.2 mM EDTA, 100 mM Tris at pH = 8.0, 0.5 mM DTT, 0.2 mM PMSF) supplemented with 0.5 mM ATP, 20 mM creatine phosphate, and 3.2 mM MgCl_2_. Reactions were incubated at 4°C for 1 h followed by electrophoresis on a 6% (w/v) nondenaturing gel. The signal was registered with radiographic film or was exposed to a phosphoimaging screen and scanned on a FLA-5100 scanner (Fujifilm).

### RNA pull-down

RNA pull-down was performed as previously described (Choudhury et al. 2014). In brief, total protein extracts from P19 or HeLa cells were incubated with in vitro-transcribed RNAs chemically coupled to agarose beads. The incubation was followed by a series of washes with buffer G (20 mM Tris pH 7.5, 135 mM NaCl, 1.5 mM MgCl_2_, 10% [v/v] glycerol, 1 mM EDTA, 1 mM DTT, and 0.2 mM PMSF). After the final wash, the proteins associated with the beads were analyzed by SDS–PAGE, followed by Western blotting.

### In vitro processing assays

Pre-miRNA substrates were prepared as previously described (Nowak et al. 2014). In brief, transcripts were prepared by in vitro transcription with [α-^32^P]-UTP. Gel-purified substrates (20 × 10^3^ c.p.m. [counts per minute], ∼20 pmol) were incubated in 30 µL reaction mixtures containing Roeder D buffer, 0.5 mM ATP, 20 mM creatine phosphate, and 3.2 mM MgCl_2._ Five microliters were aliquoted for a control and 1 µg of Dis3l2 (a kind gift from Andrzej Dziebmowski and Krystian Stodus [Lubas et al. 2013]) recombinant proteins was added to the remaining reaction mixture. Then the reactions were incubated at 37°C. The reactions were stopped after 5, 10, 20, and 40 min followed by aliquoting 5 µL and quenching on ice with 5 µL of 2× (Urea Dye [UED]), and followed by 8% (w/v) denaturing gel electrophoresis. Reactions with various amounts of Dis3l2 were performed for 10 min. The signal was registered with a radiographic film or by exposure to a phosphoimaging screen and scanning on a FLA-5100 scanner (Fujifilm).

### RNA interference

Pools of siRNAs were obtained from Dharmacon in the format of four independent siRNAs targeting different regions of the mRNA coding for the protein of interest. Four micrograms of siRNAs were delivered in two transfection events separated by 48 h using nucleofection technology (AMAXA), according to the manufacturer's instructions.

### Footprinting assays

Pre-miRNA-9 and pre-let-7a-1 substrates were synthesized by T7 in vitro transcription and were 5′ labeled with PKA, as described above. A formamide ladder was generated by incubating 2 µL of substrate (100 × 10^3^ c.p.m.) with 9 µL of F buffer (0.5 mM MgCl_2_ in 99.5% formamide [Molekula Deutschland Limited]) at 100°C for 10 min. The reaction was stopped by adding 9 µL of 2× (Urea Dye [UED]) and was placed on ice. The T1 ladder was generated by incubating 2 µL of substrate (100 × 10^3^ c.p.m.) with 2 µL of T1 2× buffer (20 mM sodium citrate, 7 M urea). One microliter of T1 at 1 U/µL was added and incubated at 55°C for 15 min. The reaction was stopped by adding 15 µL of 2× UED and placing on ice. Probes were added for cleaving the substrate RNA Pb (II) at 0.2, 0.3, and 0.4 mM, ribonuclease T1 at 0.5 U/µL, 0.25 U/µL, 0.125 U/µL, and ribonuclease V1 at 0.00075 U/µL, 0.000375 U/µL, and 0.00019 U/µL. Each reaction was prepared with 1 µL of RNA (50 × 10^3^ c.p.m.) and 7 µL of 1× structure buffer (12 mM Tris–HCl at pH = 7.5, 48 mM NaCl, 1.2 mM MgCl_2_). Samples were unfolded at 90°C for 1 min and left at RT for 5 min to refold. Two microliters of probes were incubated with 8 µL of substrate solution at RT or 37°C for 10 min. Reactions were run in the presence and absence of the recombinant Lin28a protein. For cleavage optimization, 200 ng/µL of Lin28a protein was used. In the final experiments with fixed probe concentrations, Lin28a was used in a gradient of 50, 100, and 200 ng/µL. Reactions were stopped by adding 10 µL of 2× UED and placing on ice. Samples were resolved on 10% polyacrylamide gel. The signal was registered with a radiographic film or via exposure to a phosphoimaging screen and then scanned on a FLA-5100 scanner (Fujifilm).

### Biolayer interferometry

BLI experiments were performed in 10 mM Tris, pH 8.5, 150 mM NaCl, 1 mM DTT, 0.5 mg/mL BSA, and 0.1% Tween on an Octet Red 96 instrument (ForteBio, Inc.) operating at 30°C. Streptavidin-coated biosensors bound to biotinylated pre-miRNA-9 or pre-let-7a-1 RNAs (0.125 ng/mL solutions) were exposed to different concentrations of Lin28a (with concentration series at 6.4–0.2 µM for both pre-let-7a-1 and pre-miRNA-9, repeated with a concentration series of 0.4–0.0125 µM for pre-miRNA-9 and 1.6–0.05 µM for pre-let-7a-1). Dissociation constants for wild-type and mutant binding were determined by plotting the increase in the Response Unit at equilibrium as a function of the protein concentration and fitting using nonlinear regression and in-house software.

### Preparation of recombinant Lin28a

Full-length Lin28a (AF521099) were cloned into the pETM-11 vector (EMBL-Heidelberg, Protein Expression Facility), introducing TEV protease-cleavable HisTag amino-terminal to the insert. The HisTag fusion protein was purified from the soluble fraction by nickel-affinity chromatography (Qiagen); after TEV cleavage, another nickel-affinity chromatography step was introduced to remove the cleaved His-tag, followed by gel filtration. The final protein was concentrated to 100 µM and stored in 10 mM Tris, pH 8.5, 150 mM NaCl, and 1 mM DTT.

## SUPPLEMENTAL MATERIAL

Supplemental material is available for this article.

## Supplementary Material

Supplemental Material
