# Supplementary material for: Lin28a uses distinct mechanisms of binding to RNA and affects miRNA levels positively and negatively
Source: RNA. 2017 Mar;23(3):317–32. doi: 10.1261/rna.059196.116 (PMC5311490; doi:10.1261/rna.059196.116)
Supplement: Supplemental Material [file supp_23_3_317__index.html]

Lin28a uses distinct mechanisms of binding to RNA and affects miRNA levels positively and negatively — Supplemental Material 

# Lin28a uses distinct mechanisms of binding to RNA and affects miRNA levels positively and negatively

## Supplemental Material

**Files in this Data Supplement:**

- Supplemental Material.pdf
