# Supplementary material for: Lin28a uses distinct mechanisms of binding to RNA and affects miRNA levels positively and negatively
Source: RNA. 2017 Mar;23(3):317–32. doi: 10.1261/rna.059196.116 (PMC5311490; doi:10.1261/rna.059196.116)
Supplement: Supplemental Material [file supp_059196.116_Supplemental_Material.pdf]

SUPPLEMENTARY INFORMATION

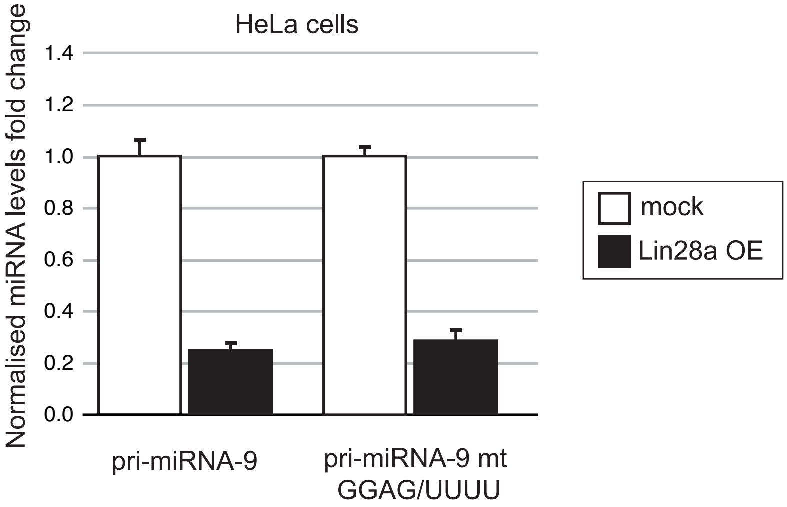

Supplementary Figure 1. Real-time qRT-PCR analysis of mature miRNAs derived from pri-miRNA-9 and pri-miRNA-9 mt expressed alone (mock) or in the presence of Lin28a (Lin28a OE). The values were normalized to miRNA-16 levels. The fold change was plotted relative to values derived from mock, which were set to 1. The mean and standard deviations (SD) of three independent biological replicates are shown.

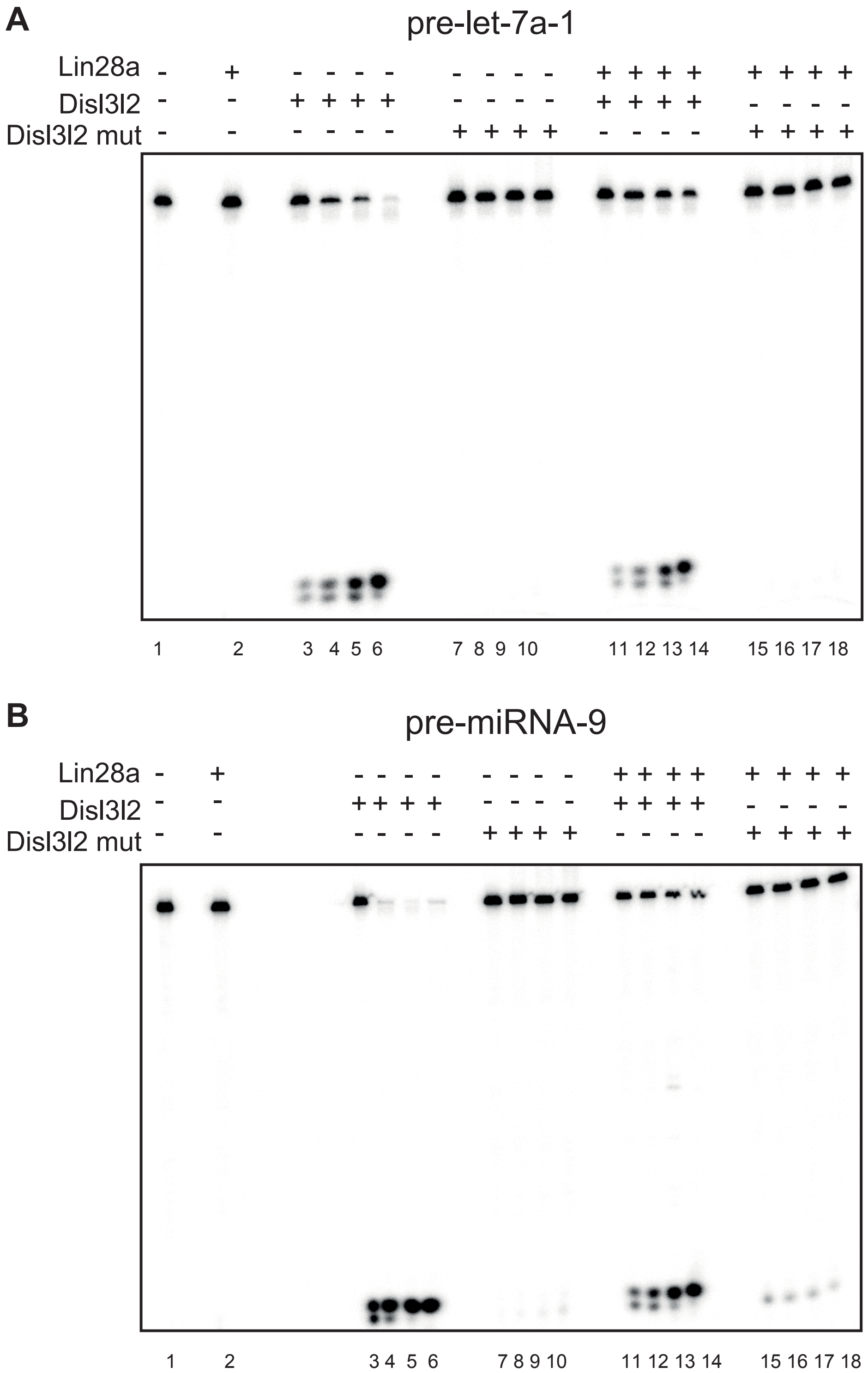

Supplementary Figure 2. *In vitro* Lin28a interferes with Dis3l2 poly(U)-independent degradation of pre-miRNAs. Internally radiolabeled pre-let-7a-1 (**A**) and pre-miRNA-9 (**B**) (3×10^3 c.p.m. (counts per minute), approximately 6 pmol) were incubated in the buffer only for 40 minutes (Lanes 1). Where indicated, 200 ng of recombinant Lin28a, Dis3l2 or catalytically dead Dis3l2 proteins was added to the reaction, which were run for 5 minutes (Lanes 3, 7, 11 and 15), 10 minutes (Lanes 4, 8, 12 and 16), 20 minutes (Lanes 5, 9, 13 and 17) and 40 minutes (Lanes 2, 6, 10, 14 and 18). The products were analyzed on an 8% denaturing polyacrylamide gel. The results are representative of two independent experiments.

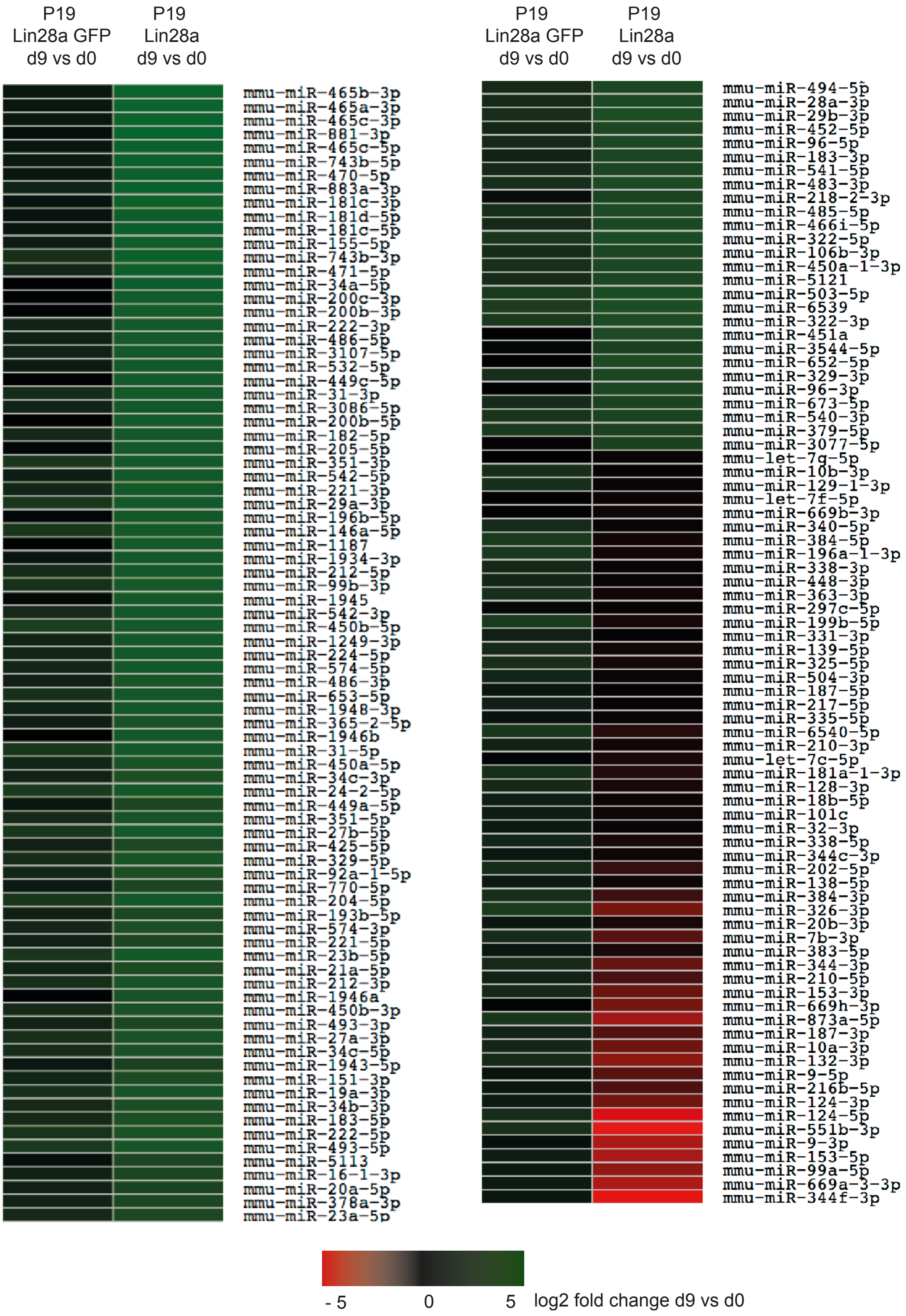

Supplementary Figure 3. Heat map of the fold change between day 0 and day 9 of neuronal differentiation of P19 cells with GFP-tagged Lin28a and of P19 cells with untagged Lin28a. The shades of green represent miRNAs upregulated more than two-fold whereas the shades of red correspond to miRNAs downregulated more than two-fold. Only miRNAs regulated more than two-fold up or down by untagged Lin28a but not regulated by GFP-tagged Lin28a are shown.

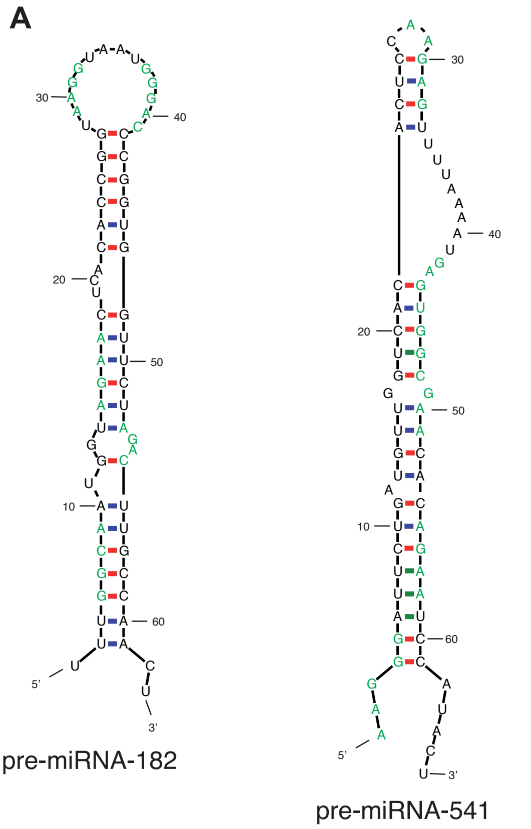
A
pre-miRNA-182
pre-miRNA-541

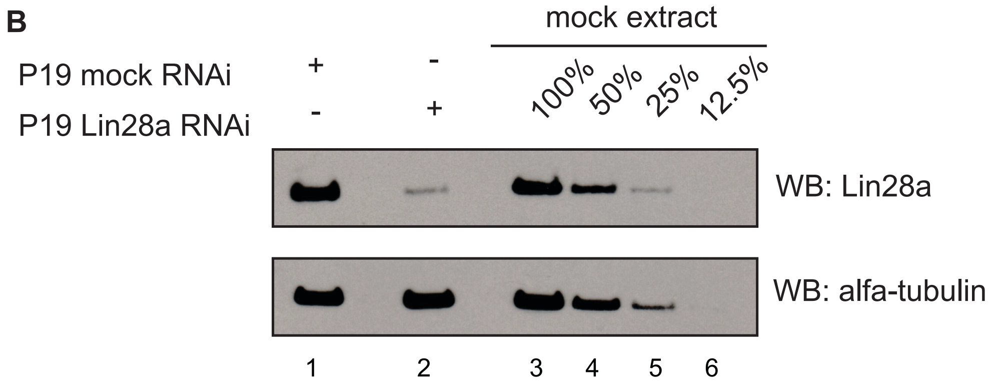
B
mock extract
P19 mock RNAi
P19 Lin28a RNAi
+
-
-
+
100%
50%
25%
12.5%
WB: Lin28a
WB: alfa-tubulin
1
2
3
4
5
6

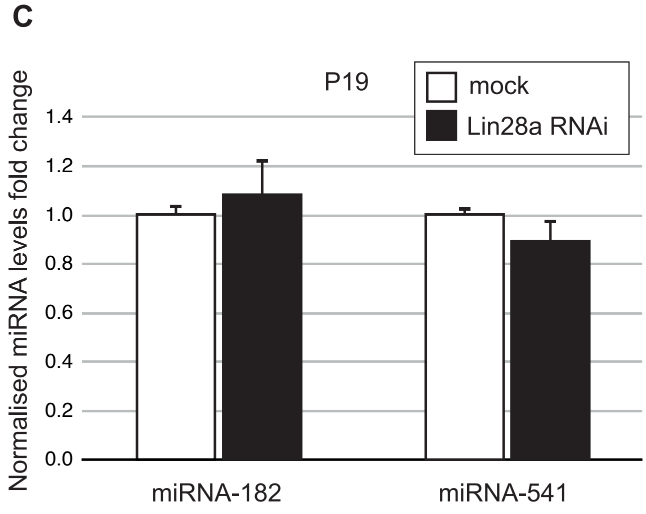
C
P19
mock
Lin28a RNAi
Normalised miRNA levels fold change
1.4
1.2
1.0
0.8
0.6
0.4
0.2
0.0
miRNA-182
miRNA-541

Supplementary Figure 4. Levels of miRNA-182 and miRNA-541 are not affected by Lin28a knock down in undifferentiated P19 cells. (**A**) Predicted secondary structures of pre-miRNA-182 and pre-miRNA-541. Putative Lin28a binding sites (based on the Lin28a CLIP motifs from Wilbert et al. 2012) are marked in green. (**B**) Western blot analysis of protein extracts from mock-depleted P19 cells (Lane 1) and Lin28a-depleted P19 cells (Lane 2). Lanes 3 through 6 show serial dilutions of total protein extracts from mock-depleted P19 cells, providing an estimation of the linearity of the western blot assay and the limit of detection. (**C**) Real-time qRT-PCR analysis of the mature miRNA-182 and miRNA-541 levels in P19 cells. The results from the mock-depleted cells are shown as white bars; the results from Lin28a-depleted cells are shown as black bars. The values were normalized to miR-16 levels. The fold change was plotted relative to values derived from mock-depleted cells, which were set to 1. The mean and standard deviations (SD) of three independent biological replicates are shown.
